# Developing a How-to-Guide for Health Technology Reassessment: "The HTR Playbook"

**DOI:** 10.34172/ijhpm.2021.180

**Published:** 2021-12-29

**Authors:** Lesley J.J. Soril, Adam G. Elshaug, Rosmin Esmail, Kalipso Chalkidou, Mohamed Gad, Fiona M. Clement

**Affiliations:** ^1^Department of Community Health Sciences, Cumming School of Medicine, University of Calgary, Calgary, AB, Canada.; ^2^Health Technology Assessment Unit, O’Brien Institute for Public Health, University of Calgary, Calgary, AB, Canada.; ^3^Centre for Health Policy, Melbourne School of Population and Global Health, The University of Melbourne, Melbourne, VIC, Australia.; ^4^Melbourne Medical School, The University of Melbourne, Melbourne, VIC, Australia.; ^5^Alberta Health Services, Calgary, AB, Canada.; ^6^International Decision Support Initiative, London, UK.; ^7^Global Health and Development Group, School of Public Health, Imperial College London, London, UK.

**Keywords:** Health Technology Reassessment, Low Value Care, Medical Overuse, Health Services Misuse, Disinvestment, De-Implementation

## Abstract

**Background:** To develop a knowledge translation (KT) tool that will provide guidance to stakeholders actively planning or considering implementation of a health technology reassessment (HTR) initiative.

**Methods:** The KT tool is an international and collaborative endeavour between HTR researchers in Canada, Australia, and the United Kingdom. Evidence from a meta-review of documented international HTR experiences and approaches provided the conceptual framing for the KT tool. The purpose, audience, format, and overall scope and content of the tool were established through iterative discussions and consensus. An initial version of the KT tool was beta-tested with an international community of relevant stakeholders (i.e., potential users) at the Health Technology Assessment International 2018 annual meeting.

**Results:** An open access workbook, referred to as the HTR playbook, was developed. As a KT tool, the HTR playbook is intended to simplify the complex HTR planning process by navigating users step-by-step through 6 strategic domains: characteristics of the candidate health technology (*The Stats and Projections*), stakeholders to engage (*The Team*), potential facilitators and/or barriers within the policy context (*The Playing Field*), strategic use of different levers and tools (*The Offensive Plays*), unintended consequences (*The Defensive Plays*), and metrics and methods for monitoring and evaluation (*Winning the Game*).

**Conclusion:** The HTR playbook is intended to enhance a user’s ability to successfully complete a HTR by helping them systematically consider the different elements and approaches to achieve the right care for the patient population in question.

## Background

**Key Messages**

** Implications for policy makers**

- The health technology reassessment (HTR) playbook is a practical and user-friendly tool that will guide policy-makers through the complex process of planning a HTR initiative.
- With this knowledge translation (KT) tool, policy-makers will systematically consider not only the candidate health technology for HTR, but also the stakeholders, the policy context, and the different levers and tools to optimize technology use.
- Policy-makers are encouraged to provide feedback on their experiences with the HTR playbook in order to refine the KT tool and create a more robust guide for policy-makers.

** Implications for the public**

Low value care is a ubiquitous issue among healthcare systems internationally. Low value care can be harmful to patients and expose them to unnecessary medical procedures and treatments. In addition, public healthcare systems are often faced with the challenge of improving the quality of healthcare delivery under fiscal constraints. Members of the public would benefit from health technology reassessment (HTR) as it is an ongoing process to maximize the value for money in the healthcare system and ultimately ensure that the right care is provided to the right patient in the right setting. The HTR playbook is intended to facilitate greater uptake of HTR practices and, in turn, promote a sustained culture of high value care for patients and optimal use of scarce healthcare resources.

Once health technologies (eg, pharmaceuticals, devices, tests, procedures) are adopted into the healthcare system there are few standard or mandatory process to continue assessing their real-world utilization and/or costs to ensure they are still providing optimal value for money.^1,2^ This can be problematic and lead to continued use of obsolete technologies, underuse of clinically effective technologies, and/or overuse or misuse of technologies that are clinically ineffective or inefficient. The latter of these examples is more commonly referred to as low value care.^3-5^ If left unaddressed, low value care can be harmful to patients and limits the delivery of efficient, high-quality, and evidence-based care.^4,6-8^

Discussions regarding health technologies are shifting from primarily evidence-informed adoption of new technologies, to evidence-informed management and optimal use of technologies throughout their lifecycle.^9^ This shift acknowledges the need to develop and implement novel approaches for technology management that span the entire technology lifecycle. Various approaches have been proposed,^10-13^ including health technology reassessment (HTR). HTR is defined as the systematic, evidence-based assessment of the clinical, economic, ethical and social impacts of an existing technology in the healthcare system to inform its optimal use relative to its alternatives.^14^ The primary objective of HTR is to support the development and implementation of evidence-informed policies and practice change to achieve optimal value for money of existing technologies in the healthcare system throughout their lifecycle.^15^

A conceptual model for HTR was developed and is composed of three broad phases: (1) *technology selection*, where candidate technologies are identified and prioritized; (2) *decision*, where evidence is synthesized and policy or practice recommendation is made; and (3) *policy action*, where the recommendation is implemented and subsequently monitored and evaluated.^16^ In addition, two foundational components, *meaningful stakeholder engagement* and *ongoing knowledge exchange and utilization*, cross all three phases.^16^ Despite this conceptual grounding, implementation of HTR initiatives in real-world policy and practice settings is complex, with practitioners often faced with methodological and practical challenges that hinder their success. This is likely owing to the dearth of information and/or tools to thoughtfully guide the planning process for a HTR,^17-20^ for instance it is unclear who to engage, what changes need to take place, and what resources are required to ensure success. To facilitate implementation of HTR initiatives, we sought to develop a knowledge translation (KT) tool to help users plan for and navigate through common complexities of HTR.^21^ The application of KT to the field of HTR has been proposed to guide and advance HTR into practice.^22^ This paper describes the development of the KT tool, referred to as the *HTR playbook*, a how-to-guide for planning a HTR initiative.

## Methods

###  Development Team

The HTR playbook was developed by a team of researchers from the University of Calgary in Canada, the University of Melbourne in Australia, and Imperial College London in the United Kingdom.Members of the development team are experts in the areas of HTA, HTR, and KT and each has previous experience leading or advising HTR and KT initiatives in their respective healthcare system contexts^23-27^ and other international contexts.^28,29^

###  Review of Evidence

A review of systematic reviews, a meta-review,^30^ was conducted to characterize the empirical experiences and approaches to HTR and related initiatives (eg, disinvestment, de-adoption, de-implementation); details of this work have been previously published.^31^ In brief, we identified several terms and ideas common to executing HTR processes and relating to: the technology value and problems with its use (eg, overuse, underuse, mis-use); policy actors and context; outcomes and measurement/evaluation; and policy levers/tools and resources. These concepts emerged from the literature illustrate not only the breadth of possible issues that relevant stakeholders (eg, healthcare system leaders, healthcare professionals, patients, government policy-makers) might face when considering implementation of a HTR, but also the guiding options that may best address the specific needs and policy issues within their contexts. The concepts identified from the meta-review provided the evidentiary foundation for the KT tool. Research ethics board approval was not applicable as this work involved only secondary data.

###  Deliberations and Pilot Testing

Members of the development team participated in an initial brainstorming session held in March 2018 to define the purpose, audience, and overall scope of the HTR playbook. Members that were not able to attend the initial brainstorming session provided input through electronic correspondence. The concepts identified from the meta-review were discussed and organized into strategic domains that would frame the KT tool. The domains were then operationalized either exercises or reflexive questions for the potential users (ie, those planning prospective HTR initiatives) to consider and address. In addition, conceptual guidance from the HTR model was also reflected in the development of the strategic domains.^16^ Lastly, pertinent terminology, guiding principles, and other resources to include for potential users were determined. Subsequent in-person and online deliberations with the development team were held over a 1-month period to finalize the content and layout of an initial draft of the KT tool (HTR playbook).

 The draft of the HTR playbook was reviewed by a group comprised of policy stakeholders from Canadian provincial ministries of health (n = 3) and regional HTA producers from Canada and Europe (n = 3); all had extensive experience either conducting, developing, and/or implementing policy decisions based on HTAs. The group was asked to provide comments on the sequence, language, coherence, and overall utility of the tool. This pilot testing was conducted to ensure that the conceptual translational into the KT tool was relevant for potential users. Stakeholder feedback was used to render the resulting ‘workbook’ more user-friendly. The final format of the KT tool was achieved through discussion and consensus with the development team.

###  Beta Testing

The HTR playbook underwent beta-testing by attendees of a pre-conference workshop entitled “The Current State of Affairs in Health Technology Reassessment” held at the Health Technology International (HTAi) 2018 annual meeting (June 2018; Vancouver, Canada). Following didactic presentations by the development team, workshop attendees were asked to breakout into groups of 5-6 and each was assigned a specific “HTR case study” characterizing a candidate technology for HTR. The case studies were developed from Choosing Wisely Canada recommendations concerning low value technologies and that should be avoided or not routinely offered for certain patient groups or clinical circumstances.^32^ Groups were then instructed to use the HTR playbook as a guide to plan a hypothetical HTR initiative. Members of the development team circulated between the groups to guide them through the exercise, help facilitate discussions, and provide clarification when needed. At the end of the exercise, feedback on ways to improve the KT tool was obtained from the attendees both orally and in writing via comments cards.

## Results

###  Emergent Concepts from the Literature

**Figure 1:**
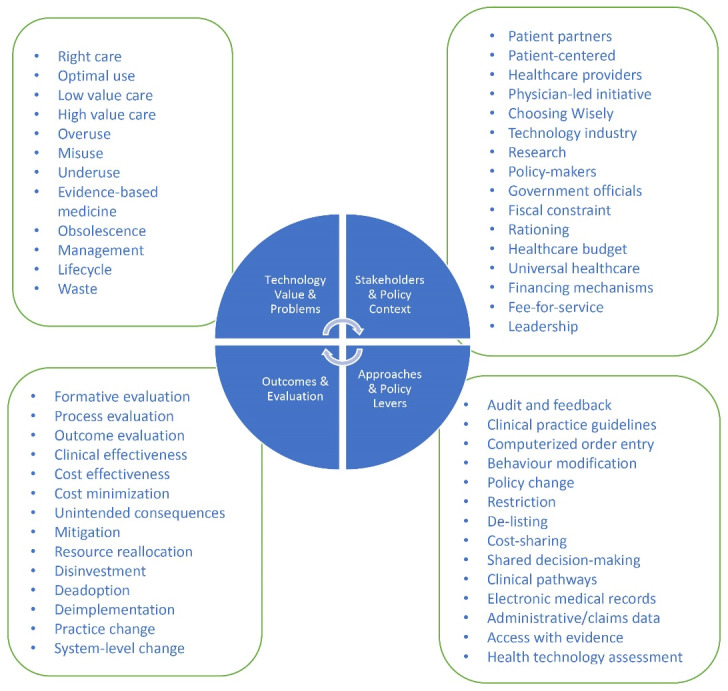
Common Concepts Identified From Meta-Review of Applied Health Technology Reassessment Literature.

The four major concepts that emerged from the literature on empirical experiences and approaches to HTR are synthesized in Figure 1. To operationalize and translate the elements within these four concepts for the KT tool, they were subsequently reorganized into the following 6 strategic domains (Table): health technology; policy stakeholders; policy context; unintended consequences; approaches and policy levers; evaluation. The 6 strategic domains represent integral components to a HTR planning process that should not only be considered, but also well-understood and accounted for in advance of commencing a HTR initiative.

**Table:** Description of Strategic Domains in HTR Planning Process

**Strategic Domains**	**Purpose**	**Facilitated Activity in the Knowledge Translation Tool**
Health technology	Understand the characteristics of the candidate technology for HTR, including how it is currently used and defining its optimal use	Characterize what the technology is, who it is being used for (ie, patient group), where it is being used, and how it is being delivered and/or paid for Based on the above information, users reflect on the current value of the technology, the utilization gap, and the anticipated outcomes of a HTR in order to specify a goal for their HTR initiative
Policy stakeholders	Recognize the different stakeholders that should be engaged in the HTR initiative	Identify stakeholders to engage in the HTR initiative from groups such as patients, healthcare professionals, healthcare system leaders, government policy-makers, industry representatives, and academic and other researchers In addition to the users own role, the various interests, role(s) of and/or level of engagement for each stakeholder group should be outlined
Policy context	Recognize key features of the healthcare system context and how they may serve as facilitators and/or barriers for the HTR initiative	Outline the different financing modalities and the organization of the healthcare system, the roles of the government and/or other payers Identify potential political forces or issues and contextual assets available in the healthcare system context
Consequences	Prepare for unintended consequences that may arise from actions taken in the HTR initiative	Reflect on all potential positive or negative unintended consequences that may result from implementation of the selected approaches and/or policy levers If necessary, users should describe potential counter-actions to mitigate these consequences
Approaches and policy levers	Determine specific action(s) required to achieve optimal technology use	Select appropriate approaches and policy levers at the healthcare delivery, financial, governance levels to implement as part of the HTR initiative Consider the ideal conditions (ie, stakeholder involvement and available assets) for implementation of each option
Evaluation	Plan the final evaluation of the HTR initiative	Specify what outcome(s) will be evaluated, how evaluation will be conducted, and when evaluation will take place Remind users of their intended goal and that they can change their course of action in order to adapt to evolving circumstances and problems

Abbreviation: HTR, health technology reassessment.

###  Strategic Domains

####  Health Technology

As an initial step, stakeholders undertaking HTR implementation need to begin with an understanding of the characteristics of the candidate health technology to reassess; this include characterizing how the technology is currently used (ie, overused, misused, or underused), the issues surrounding that use (ie, current value and utilization gap), and the outcomes anticipated from the HTR (ie, increased use, decreased use, no change, or complete exit of the technology from the healthcare system). With this information in mind, stakeholders are able to specify the overall goal(s) of the HTR initiative and plan next steps in the subsequent strategic domains. This goal should be framed around what is considered optimal use of the candidate technology^31^ based on best available evidence.

####  Policy Stakeholders

The other stakeholders or groups of stakeholders that should be engaged and actively involved in the conduct of the HTR initiative may include: patients, community, and civil society organizations; healthcare professionals; industry representatives; healthcare system leaders; government policy-makers; and academic and other researchers.^4,31^ In addition to identification of stakeholders, the roles and interests of the other stakeholders need to be articulated as part of the engagement process. Additionally, the lead should identify what their own role(s) and interest(s) are within the group. An interdisciplinary team is strongly recommended, engagement of a stakeholder group for the sole purpose of representation is insufficient; engagement must be authentic. Thus, thoughtful consideration must be applied when engaging stakeholders. A preliminary phase could possibly be envisaged to adequately involve and inform stakeholders in the assessment process.^16^

####  Policy Context

There are a number of features of the policy context or healthcare setting that can serve as barriers or facilitators for an HTR initiative. These may include the organization and governance of the healthcare system, the health insurance and financing mechanisms, the political forces or issues, and the analytic assets and infrastructure available at the users’ disposal.^33^ The lead stakeholder must recognize and account for such features in order to appropriately frame the subsequent strategies to optimize use of the candidate technology under reassessment. Understanding the KT field, particularly the KT theories, models, and frameworks that may be relevant to HTR, may also be of value to stakeholders.^34^ The use of process models, for example, can provide structure to systematically assess barriers and facilitators to implementation.

####  Unintended Consequences

The overall goal of a HTR, specified at the outset of the planning process, is an intended consequence of the initiative. Unintended consequences—positive or negative—however, may also arise from particular actions taken in a HTR. For example, one potential negative unintended consequence that could occur from removing one low value technology from practice is the increased use of another technology that is potentially less effective and/or more expensive.^15,16^ The lead stakeholder must outline potential unintended consequences, then articulate and plan the actions they would take to monitor and mitigate them. This step requires broad consideration of not only the effects on the candidate technology itself, but also the intended and unintended effects to all relevant stakeholders across the framed policy setting.

####  Approaches and Policy Levers

International recommendations for policy levers to address issues of technology underuse and overuse are published and categorized as either delivery arrangements, financial arrangements, and governance arrangements.^4^ In the context of a HTR, multiple approaches or policy levers within and between these three categories may be applied to target change at various levels of the policy context.^31^ Success with such approaches and levers, in terms of achieving the desired goal of the HTR, may require extensive stakeholder engagement and resources (eg, data assets). As such, the lead stakeholder will need to broadly consider what assets are available in a given policy context to determine the feasibility of implementing specific approaches or levers for a HTR initiative.

####  Evaluation

Evaluation can incorporate formative, process, and outcome evaluation using both quantitative and qualitative methods.^16^ Further, clarity on *what* will be evaluated (eg, technology use and cost), how to evaluate (eg, what data source, what human resources are needed, what evidence to compare to), and when the evaluation will take place (eg, at what point and over what period of time) must be considered at the outset of planning a HTR initiative. Continual monitoring and appropriate evaluation, once a HTR initiative has begun, will give users the ability to remain nimble and responsive to stakeholders and evolving circumstances. Importantly, if the desired outcomes are not achieved, the lead stakeholder should assess the need to pivot their direction considering an alternative course(s) of action.

###  The KT Tool: The HTR Playbook

The final version of the KT tool was entitled the “HTR playbook” (Supplementary file 1). A fillable workbook is available as an open-access document through the HTA Unit, University Calgary website (https://obrieniph.ucalgary.ca/sites/default/files/teams/5/HTR%20Playbook%20-%202021-01-06%20(1).pdf).

 The anticipated users of the HTR playbook (the lead stakeholder[s]) include individuals or groups actively planning or considering implementation of a HTR initiative and are either in a position to directly realize change in policy and/or practice, or able to partner with the appropriate stakeholders to do so. A HTR initiative can, therefore, be viewed as both a ‘bottom-up’ approach, wherein healthcare professionals and patient and community groups independently seek to initiate efforts that will improve the quality and safety of care, as well as a ‘top-down’ approach, incited by policy-makers and healthcare system leaders as they face the challenge of ensuring safe and high-quality care within a climate of scarce healthcare dollars.

**Figure 2:**
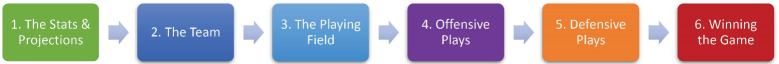
Plain Language Description of the Strategic Domains in the Health Technology Reassessment Playbook.

To render the ‘workbook’ more user-friendly and accessible for a wider audience, analogies were made between colloquial sports or coaching language and the concepts and strategic domains of the HTR planning process. For instance, a set of guiding principles for HTR^16^ are referred to as *League Rules* and are outlined at the outset of the HTR playbook to help ground users as they navigate the planning process. While HTR can look quite different between healthcare system contexts and for different technologies,^16^ the principles that guide and the language used within those processes are likely to be quite similar. In addition, relevant technical definitions,^4^ described in the playbook as *Chalk Talk*, are also provided to orient users to the language adapted in the field. Furthermore, the six strategic domains in HTR planning processes were translated in the playbook as: *The Stats and Projections* (the health technology, including its current use relative to its optimal use), *The Team* (the stakeholders), *The Playing Field* (the policy context), *The Offensive Plays* (the approaches and policy levers), *The Defensive Plays* (the unintended consequences), and *Winning the Game* (the metrics and methods for evaluation) (Figure 2).

####  How to Use the HTR Playbook

Users of the HTR playbook can begin by orienting themselves with the *League Rules* and the *Chalk Talk*;these sections are purposefully provided for users at the outset to help ground their thinking. It is assumed that users have already identified and prioritized a candidate technology for HTR and thus can next complete the ‘workbook’ phase of the HTR Playbook by addressing the 6 strategic domains, which are also organized in the order in which they are recommended to be completed. For each domain, several guiding questions and/or exercises are posed to users and are meant to incite reflection on the needs and potential gaps to ensure a successful HTR. The responses indicated by users will clarify their actions required in their planning processes. As the planning and implementation phases of an HTR progress, users can revisit any of the strategic domains and make adjustments to “switch up the plays” in order to overcome any evolving challenges or barriers. The HTR playbook does not indicate the specific timing at which an adjustment may be warranted, users must make this decision at their own discretion. Lastly, given that various stakeholders may be engaged at different moments, and may not all contribute to the initial planning, it is recommended that all stakeholders at minimum are provided an opportunity to review the final responses in the playbook.

###  Feedback From Beta Testing

 There was a total of 30 international workshop attendees from across North America (ie, Canada and the United States), Australia, Europe, the United Kingdom, South America, and Asia. This group included national (n = 9) and regional HTA producers (n = 3), government and health system payers (n = 3), academic researchers (n = 6), and representatives from industry (n = 9). Thus, all attendees had either direct experience conducting and contributing to HTAs, or were familiar with the HTA process. While most had limited prior knowledge and/or experience with HTR, all of the attendees expressed interest in learning about or leading HTR initiatives in their respective healthcare system contexts and, as such, were possible users of the HTR playbook. From the group exercise, attendees found the HTR playbook to be useful for evaluating the case studies and appreciated the sports analogies used throughout the tool. Further, the framing and ordering of the HTR planning process, in the 6 strategic domains, made the endeavour of planning the hypothetical HTR more transparent and approachable for the attendees. Most attendees found the guiding questions to provoke thoughtful group discussions. However, all of the breakout groups experienced difficulty answering one or more of the guiding questions and attributed such challenges to their current limited experience with HTR and unfamiliarity with the common terminology. Many attendees expressed appreciation for having ‘in-person’ training of the HTR playbook through the workshop exercise and likelihood of future use.

## Discussion

HTR is gathering international momentum. There is increasing acknowledgement that mechanisms to enable lifecycle management of health technologies, such as HTR, is required to ensure their ongoing optimal use.^3-7^ This notion is also in line with the principles of value-based healthcare, which is predicated on the allocation and reallocation (ie, through disinvestment) of healthcare resources to maximize value from the individual, technical, allocative, and societal perspectives.^8,35^ Therefore to build on this momentum, we developed the HTR playbook as a KT tool to further the practice of HTR. The objective of the playbook is to simplify the complex process of planning and implementing a HTR initiative by navigating users step-by-step through 6 strategic domains. While the KT tool helps to frame pertinent issues in the HTR planning process, it should not be viewed as a rigid recipe; in fact, the depth of information presented is intended to encourage users to think broadly and inclusively in their planning processes. Given that healthcare environments vary widely, the HTR playbook also highlights how the evidence, stakeholders, and resources required to achieve a successful HTR initiative must be customized to the individual healthcare context.^16,31^

A strength of the HTR playbook is that it was designed for broad application. HTR should not be viewed as an exclusively top-down nor bottom-up approach, but rather a collaborative endeavour involving diverse individuals and/or groups that may be impacted by the outcome of the HTR.^31^ Therefore, the HTR playbook was developed to empower users to design and implement HTR initiatives from where they stand in the healthcare system. HTA producers and organizations, for example, are well-positioned to promote and/or directly implement initiatives and tools related to HTR with the aim of disinvesting from technologies of low value in order to reinvest in higher value care.

From a practical perspective, the questions and exercises posed throughout the playbook can also help users evaluate whether there is necessary and sufficient information, support, and/or resources to go forward with a HTR initiative. An unanswered question in any of the 6 strategic domains could indicate a weakness or paucity and should signal to a user that a HTR should not be initiated until which time the issue(s) is addressed. For instance, if users do not know what basic data assets they have at their disposal to monitor and evaluate outcomes (eg, electronic medical records, prospective registry databases) they cannot appropriately plan their *Offensive* nor *Defensive* plays.

It is worth noting that the HTR playbook is not intended to help users determine which technology to reassess; use of this KT tool assumes that an existing technology has already been identified and prioritized over other candidates through transparent, systematic, and evidence-informed means. Several resources for and approaches to candidate technology selection have been previously described^23,25,26,36^ and we recommend that users consider these in conjunction with the HTR playbook. Published lists of low value technologies, such as from the international Choosing Wisely campaign,^37^ offer an efficient starting ground to help identify candidates for HTR as the listed technologies have previously been classified as low value for certain patients and in certain circumstances.^23^ In addition, indicators to measure use and costs of technologies on the low value lists, through claims and administrative health data for example, can help to prioritize technologies for HTR using criteria of variability (eg, provider, geographic, temporal), volume, and/or budgetary impact.^23,25,26,36^

 A limitation of the HTR playbook is that is has not been applied in a real-world policy context. Using hypothetical HTR case studies, the beta testing exercise at our HTAi 2018 pre-conference workshop offered initial learnings of the tool’s perceived utility among a convenience sample of users. However, ongoing application by different users and in different contexts is required to appropriately elucidate the utility of the KT tool in practice as well as identify further areas for improvement. Real-world application of the HTR playbook would assess how robust the KT tool is at guiding HTR initiatives for both low and high value technologies. Further, use of the KT tool in policy contexts with market-based healthcare arrangements, as compared to those with publicly-funded health insurance coverage, could highlight key conceptual and operational differences in HTR. If the policy context was in the United States, for example, would the insurance companies or healthcare organizations be best positioned to lead a HTR initiative? Moreover, how would those leading HTR initiatives reconcile the possible decrease in revenue caused by the elimination of currently used technologies? Ultimately, by providing electronic and open access to the HTR playbook, we hope to facilitate broad uptake to and solicit feedback to refine the KT tool from international users actively planning or considering implementation of HTR initiatives.

## Conclusion

 The HTR playbook was designed with an international perspective, informed by empirical evidence and experiential knowledge of international HTA, HTR, and KT experts. It is intended to enhance a user’s ability to systematically consider not only the candidate technology for HTR, but also the stakeholders, the policy context, and the different levers and tools to achieve the right care for the patient population in question. Tools like this one are a critical step towards successful implementation of a HTR initiative.

## Acknowledgements

 We gratefully acknowledge the efforts of the policy stakeholders and HTA producers who critically reviewed the draft KT tool. We also thank the attendees of the HTAi pre-conference workshop involved in the beta-testing of the HTR playbook.

## Ethical issues

 Research ethics board approval was not applicable as this work involved only secondary data.

## Competing interests

 Authors declare that they have no competing interests.

## Authors’ contributions

 Design of the study (LJJS, FMC, AGE, MG, KC); acquisition and management of data (LJJS, FMC), analysis and interpretation of data (LJJS, FMC); preparation of manuscript (LJJS, FMC); review of manuscript (LJJS, AGE, RE, MG, KC, FMC); approval of manuscript (LJJS, AGE, RE, MG, KC, FMC).

## Supplementary files

Supplementary file 1. The HTR Playbook.
