# Supplementary material for: Developing a How-to-Guide for Health Technology Reassessment: "The HTR Playbook"
Source: Int J Health Policy Manag. 2021 Dec 29;11(11):2525–32. doi: 10.34172/ijhpm.2021.180 (PMC9818092; doi:10.34172/ijhpm.2021.180)
Supplement: Supplementary file 1 — The HTR Playbook. [file ijhpm-11-2525-s001.pdf]

Article title: Developing a How-to-Guide for Health Technology Reassessment: "The HTR Playbook"

Journal name: International Journal of Health Policy and Management (IJHPM)

Authors' information: Lesley J.J. Soril[1,2], Adam G. Elshaug[3,4], Rosmin Esmail[1,5], Kalipso Chalkidou[6,7], Mohamed Gad[6,7], Fiona M. Clement[1,2]*

[1]Department of Community Health Sciences, Cumming School of Medicine, University of Calgary, Calgary, AB, Canada.

[2]Health Technology Assessment Unit, O'Brien Institute for Public Health, University of Calgary, Calgary, AB, Canada.

[3]Centre for Health Policy, Melbourne School of Population and Global Health, The University of Melbourne, Melbourne, VIC, Australia.

[4]Melbourne Medical School, The University of Melbourne, Melbourne, VIC, Australia.

[5]Alberta Health Services, Calgary, AB, Canada.

[6]International Decision Support Initiative, London, UK.

[7]Global Health and Development Group, School of Public Health, Imperial College London, London, UK

(*Corresponding author: fclement@ucalgary.ca)

Supplementary file 1. The HTR Playbook

I. HOW TO USE THE HTR PLAYBOOK

Policy decisions concerning health technologies have shifted from primarily evidence-informed adoption of new technologies, to evidence-informed management of technologies throughout their lifecycle. Various policy approaches have been proposed internationally to support ongoing management of technologies, including that of Health Technology Reassessment (HTR). HTR is defined as the systematic, evidence-based assessment of the clinical, economic, ethical and social impacts of an existing health technology (i.e., pharmaceutical, device, test, procedure, etc.) in the healthcare system to inform its optimal use relative to its alternatives.

This HTR playbook is a starting point for all stakeholders engaged in the HTR process. Based on documented international experiences, this playbook provides a step-by-step guide on how to plan, implement and evaluate a successful HTR. We include 6 strategic domains:

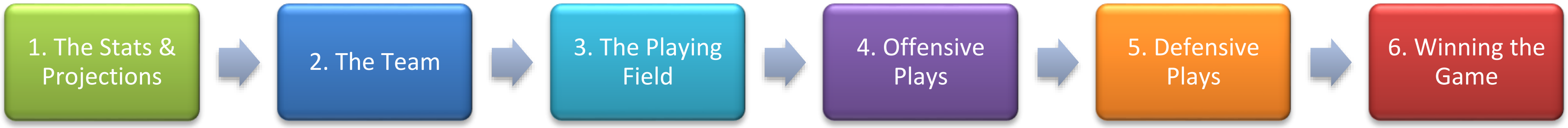

Within each domain a number of guiding questions are posed; these questions are meant for you to reflect on the information you need to know and/or acquire for your HTR initiative. The information presented in this playbook is intended to enhance your understanding of how to address issues of overuse, misuse or underuse of existing health technologies. Importantly, this may guide strategic use of different levers and tools to achieve the right care within your context from where you stand.

Contributors

Lesley J.J. Soril, PhD; Rosmin Esmail, PhD; and Fiona M. Clement, PhD on behalf of the Health Technology Assessment Unit
O'Brien Institute for Public Health and Cumming School of Medicine, University of Calgary

Adam G. Elshaug, PhD
Melbourne School of Population and Global Health, University of Melbourne

Mohamed Gad, MD, MA and Kalipso Chalkidou, MD, PhD
International Decision Support Initiative; Global Health Development Group, Imperial College London

II. CHALK TALK

Key Words	Definitions (derived and adapted from Elshaug *et al.,* 2017 and the *Right Care series* published in the Lancet)
Low Value Care	A health service, treatment or procedure in which evidence suggests it confers no or very little benefit for patients, or risk of harm exceeds probable benefit, or, more broadly, the added costs of the intervention do not provide proportional added benefits[1]
High Value Care	A health service, treatment or procedure in which evidence suggests it confers benefit on patients, or probability of benefit exceeds probable harm, or, more broadly, the added costs of the intervention provide proportional added benefits relative to alternatives
Right Care	Care that is tailored for optimising health and well-being by delivering what is needed, wanted, clinically effective, affordable, equitable, and responsible in its use of resources
Overuse	Provision of a service, treatment or procedure above its intended scope of use; unlikely to increase the quality or quantity of life, that poses more harm than benefit, or that patients who were fully informed of its potential benefits and harms would not have wanted
Underuse	Failure to deliver a service, treatment, or procedure that is highly likely to improve the quality or quantity of life, that represents good value for money, and that patients who were fully informed of its potential benefits and harms would have wanted
Misuse	Provision of a service, treatment or procedure outside of its intended or funded scope of use; unlikely to increase the quality or quantity of life, that poses more harm than benefit, or that patients who were fully informed of its potential benefits and harms would not have wanted

III. LEAGUE RULES

We outline a set of eight overarching principles to guide the development of your HTR process. These are informed by a review of the literature, an environmental scan, and expert input and also serve as backdrop for the recommendations and policy levers discussed in this playbook.

1. ***The HTR process must be context-specific and flexible, with an expectation that it could evolve over time***
2. ***Stakeholders must be meaningfully engaged and ideally embedded within any HTR process***
3. ***HTR requires high level political support***
4. ***Feasibility assessment, done collaboratively with stakeholders, must be done early in the HTR process***
5. ***HTR is best integrated with other evidence-informed decision-making processes, such as the development of clinical practice guidelines and/or clinical care pathways, and overall quality improvement initiatives***
6. ***HTR should be viewed as a broadening of the scope of traditional health technology assessment (HTA) processes***
7. ***Evidence generated from the HTR process should be conceptualized as mode 2 knowledge (i.e., generated in context for a practical purpose)***
8. ***Monitoring and evaluation are essential and must be flexible and robust enough to capture unintended consequences***

#1. THE STATS & PROJECTIONS

Understanding characteristics of the health technology itself, including the way in which it is currently being used and the surrounding issues, will help you understand your next steps and the objective or goal of the HTR. Complete the following sections concerning the characteristics, value, and utilization gap of the technology and outcomes of its reassessment. Use this information to then specify your goal for the HTR.

HEALTH TECHNOLOGY is a term used to describe an intervention that may be used to promote health, to prevent, diagnose or treat acute or chronic disease, or for rehabilitation. Health technologies include pharmaceuticals, devices, procedures and/or organizational systems used in healthcare.

What

What is the health technology being reassessed?

____

____

Who

Which patients are being treated with it?

____

____

Where

Where is this health technology being used (e.g., in-hospital, primary care physician offices)?

____

____

How

How is this care being delivered and how is it being paid for (e.g., fee-for-service, bundled payment)?

____

____

VALUE of a health technology is the impact and outcomes it achieves in relation to its cost. Value can be considered from the patient, healthcare system or societal perspectives. The spectrum of technology value spans from high value (highly beneficial, acceptable costs); low value (minimal benefit, cost irrespective); no demonstrable value (ineffective); or harmful.

What is the value of the technology? *Indicate whether it is low, high, of no demonstrable value, or harmful.*

____

UTILIZATION GAP is the difference between the optimal and observed use of the health technology. This includes overuse of a technology that is ineffective or harmful, overuse or misuse of an effective technology outside of its intended scope of use (i.e., appropriate technology, but wrong patient, indication, or time), or underuse of a technology of proven clinical- and/or cost-effectiveness. The gap may be identified through temporal, provider, and/or geographic variations in practice.

What is the utilization gap? *Indicate whether it is overused, misused, or underused.*

____

OUTCOME refers to the anticipated impacts of reassessing the technology. Think broadly in terms of outcomes, including clinical endpoints (e.g., patient safety, quality of life, satisfaction), health system or process-related outcomes (e.g., reduced length of stay), and cost savings to the health care system.

What is the anticipated outcome(s)?

____

Your goal is to ______________________________ **of** _________________________________ **for** _________________________________.
(insert optimal use) (insert health technology) (insert patient group)

#2. DRAFTING YOUR TEAM

Meaningful and broad stakeholder engagement is fundamental to the HTR process. The specific individuals that need to be engaged will depend on your healthcare context and technology in question; not all stakeholders are required at all times. Examples of five key stakeholder groups are provided below. Based on these groups, identify your role, the key players and their roles and interests in the HTR initiative.

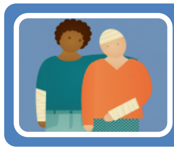

Patients, community, civil society organisations

- Present and past patients and the wider public; represented as individuals or groups (e.g., patient advocay groups with experience with technology)

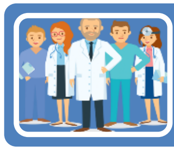

Clinical professionals

- Individuals involved in the care of patients and use of the technology; represented as individuals or in groups by clinical professional associations

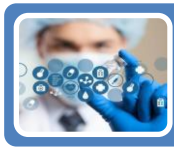

Industry representatives

- Includes technology manufacturers, pharmaceutical industry, and industry union

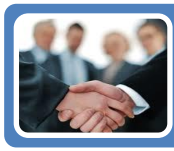

System leaders

- Administrators and executives in arm's-length (e.g., safety and health quality commissions) or non-government organisations, and thir party payers or insurers

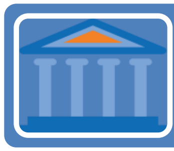

Government policy-makers

- Elected officials (e..g., Ministers of Health) at the regional (e.g., municipal, provincial, state) or federal levels

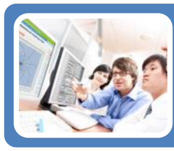

Academic and other researchers

- With expertise in health technology assessment, health economics, health services research, epiemiology, implementation science

Your role:

Your team:	Their roles:	Their interests:

#3. THE PLAYING FIELD

Describe the key features of the healthcare system context, including the organization and governance, the financing mechanisms, the political forces or issues, and the resources and analytic assets at your disposal to conduct the HTR.

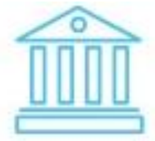

What is the role of the government and other third party payer(s)?

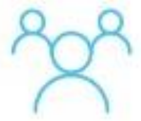

Who is covered and how is insurance financed, including any rules/limits?

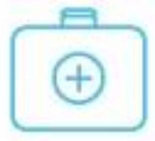

How is the delivery system organized and financed?

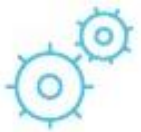

What important political forces or issues need to be considered?

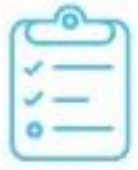

What assets are at your disposal? What is your timeline and scope?

- *Health data sources (e.g., clinical registries, administrative data)*
- *Electronic medical records*
- *Human resources (e.g., HTA or HTR analysts)*

#4. THE OFFENSIVE PLAYS

With the stats and projections, the team, and the playing field in mind, it is now time to plan out the offensive plays for your HTR initiative. Here are 3 categories of approaches and policy levers for you to consider. In order to reach your goal, you may require a variety of levers to target different stakeholder groups in your context. Therefore, we also provide recommendations for the ideal players and assets that need to be in place to employ each lever type.

Which lever is for you?	Selected Examples	Recommended When
☐**Delivery**	• Redesign workflow to encourage or facilitate optimal use of technology<br>• Adapt or implement models of care<br>• Support for physicians, including:<br>○ Best practice guidelines with do and don't do recommendations<br>○ Optimal use criteria with measurement and reporting (i.e., audit and feedback)<br>○ Electronic decision-support tools (e.g., computerized orders and alerts)<br>○ Education on evidence and tools (e.g., shared-decision-making [SDM])<br>• Support for patients, including education and tools (e.g., SDM)	*You have these players:*<br>• Patients, community, and civil society organisations<br>• Clinical professionals<br>• System Leaders<br>• Academic and other researchers<br>*You have these assets:*<br>• Health information technology, electronic health records<br>• Data on technology use and costs (e.g., claims data), and patient outcomes (e.g., registry data)<br>• Data analysts<br>• Funding
☐**Financial**	• Remove or restrict coverage or reimbursement, including restriction by indications, coverage with evidence, for guideline adherence, or value-based insurance designs<br>• Reference coverage to rate of least costly alternative and/or provider<br>• Displace coverage of existing technology when new comparator adopted<br>• Global budget with incentives and disincentives, including:<br>○ Risk sharing<br>○ Pay-for-performance<br>○ Bundled payments	*You have these players:*<br>• Patients, community, and civil society organisations<br>• Clinical professionals<br>• Industry representatives<br>• System Leaders or government policy-makers<br>• Academic and other researchers<br>*You have these assets:*<br>• Data on technology use and costs, and patient outcomes<br>• Infrastructure and human resources with expertise in HTA/HTR<br>• Funding
☐**Governance**	• Mandatory review of all new and existing technologies, regardless of how they were introduced<br>• Restriction of technology use (or providers) to centres of excellence<br>• Assign or change authority over:<br>○ Policies for eligibility of coverage, breadth of services covered, and cost-sharing for specific populations<br>○ Commercial issues concerning licensing or registering, pricing, marketing, selling and purchase products<br>○ Professional requirements for health professional training, licensure, and continuing education	*You have these players:*<br>• Clinical professionals<br>• System Leaders<br>• Government policy-makers<br>• Academic and other researchers<br>*You have these assets:*<br>• Data on technology use and costs, and patient outcomes<br>• Infrastructure and human resources with expertise in HTA/HTR<br>• Funding

#5. THE DEFENSIVE PLAYS

It is important to acknowledge that despite your best efforts, there may be some unanticipated consequences (either positive or negative) to your offensive play. For example, one potential negative unanticipated consequence that can arise from the removal of an existing technology is the increased use of another technology that is potentially less effective and/or more expensive. Consider potential unintended consequences and the actions you would take to monitor and mitigate them.

Unintended Consequence	How will you monitor for this?	How will you mitigate this?
**Unintended Consequence #1**		
**Unintended Consequence #2**		
**Unintended Consequence #3**		
**Unintended Consequence #4**		

#6. WINNING THE GAME

Evaluating the outcome(s) of your HTR initiative, against your intended goal, will help you determine if you achieved success. Evaluation can incorporate formative, process, and outcome evaluation using both quantitative (e.g., analysis of technology utilization and costs) and qualitative (e.g., observation, interviews, focus groups) methods. We offer you some key questions to reflect upon to plan your evaluation.

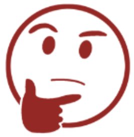

REMIND YOURSELF OF THE GOAL:

____

____

What

What will you evaluate?

____

____

____

How

How will you evaluate?

____

____

____

When

When will you evaluate?

____

____

____

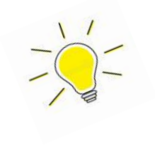

REMEMBER TO PIVOT IF NECESSARY!

You can always shake up the plays if something is not working.
